# The Swedish version of the STarT MSK Tool: cross-cultural adaption, test–retest reliability, and aspects of validity

**DOI:** 10.1186/s12891-023-06771-6

**Published:** 2023-08-10

**Authors:** Eva Rasmussen-Barr, Maria Sövelid, Rasmus Krantz, Jonathan C. Hill

**Affiliations:** 1 Department of Neurobiology, Care Sciences and Society, Division of Physiotherapy, Karolinska Institutet, Alfred Nobels Allé 23, 141 83 Huddinge, Sweden; 2 School of Medicine, Keele University, Staffordshire, UK

**Keywords:** Rehabilitation, Risk, Stratification, Musculoskeletal

## Abstract

**Background:**

Musculoskeletal disorders (MSDs) are a common reason for seeking primary health care. The STarT Musculoskeletal (MSK) tool is designed to stratify patients suffering from MSDs to risk groups, based on prognostic factors.

**Aim:**

The aim was to translate and cross-culturally adapt the STarT MSK tool in a Swedish primary health care context through testing of reliability and construct validity.

**Methods:**

We included consecutive patients with MSDs seeking primary care (*n* = 99). The STarT MSK was translated using international recommendations. Construct validity was investigated by correlation analysis (Spearmans Rho) with the following reference instruments: the Örebro Musculoskeletal Pain Questionnaire (ÖMPQ), the EuroQol 5-dimension (EQ-5D) and the Musculoskeletal Health Questionnaire (MSKHQ). Reliability was tested using test–retest (Intra Class Correlation, ICC_2.1_) (*n* = 31). Known-groups validity was calculated with a difference of 10% between risk groups based on how the participants had answered.

**Results:**

The STarT MSK was successfully translated into Swedish. The participants were grouped into low risk (*n* = 28), medium risk (*n* = 60) and high risk (*n* = 11). The construct validity showed a moderate to high correlation with the ÖMPQ (*r* = .61), EQ-5D (*r* = .59) and MSK-HQ (*r* = .56). All separate items except item 2 and 9 correlated according to predefined hypotheses. Test–retest demonstrated an excellent reliability for the total score (ICC_2.1_ 0.85) (*n* = 31). The STarT MSK tool was able to differentiate by 10% between the risk groups, based on how the participants had answered.

**Conclusion:**

The STarT MSK has been successfully translated and adapted into Swedish and shows acceptable measurement properties regarding test–retest reliability and aspects of validity and seems to be able to discriminate between the proposed risk groups. The tool can therefore be useful in a Swedish primary health care context. A future study needs to determine the tools predictive validity and to investigate if stratification to risk groups leads to a faster recovery and to lower health care costs.

## Introduction

Over the last 30 years, musculoskeletal disorders (MSDs) have become an increasingly significant factor in disability adjusted life years globally (DALYs) [1]. MSDs are also one of the most common reasons for seeking primary health care [2]. Due to an aging population and the steady increase in the prevalence of MSDs across all age groups, the impact and demands of these problems are expected to rise [3].

Many countries offer direct access to physiotherapists, meaning that physiotherapists often are the first health professionals to encounter people seeking primary health care for MSDs [4]. This entails a responsibility to target treatment or to provide a referral to other health professionals when warranted. One contributor to the burden on individuals and society is said to be poor quality healthcare, described as an inability to provide patients with appropriate care [5, 6]. In the clinical reasoning process, patient-reported outcome measures (PROMs) are therefore recommended but not always used due to various barriers [7–10]. PROMs are defined as “any report of the status of a patient's health condition that comes directly from the patient, without interpretation of the patient's response by a clinician or anyone else” [11]. There are a variety of PROMs that measure different domains, and most PROMs are lengthy, which sometimes makes them difficult for health professionals and patients to use [12]. Shorter PROMs that include measurements of various domains are therefore appreciated both by clinicians and patients, and several are recommended, such as the Core Outcome Measure Index and the STarT Back Screening Tool [13–16]. One approach used to assist clinical decision-making, thus increasing clinical effectiveness in care, is the use of a stratification instrument [13, 17–19] The STarT Back Screening Tool is such an instrument, which is designed to screen primary care patients suffering from low back pain (LBP) based on prognostic factors for the risk of a poor outcome (low, medium, and high risk) [13]. Compared to best current practice and usual primary care, the tool has demonstrated good outcomes when used in clinical settings to match different treatments with subgroups of patients suffering from LBP [13], even if a recent study reported that the STarT Back Screening Tool has a limited value when predicting future disability [20].

Although musculoskeletal conditions are usually recognized by anatomical location and associated features (e.g. impact on physical function), research suggests that pain problems in musculoskeletal areas such as the back, neck, shoulder and knee, as well as pain that co-occurs in different body regions (multi-site pain), seem to share similar underlying mechanisms and prognostic factors [21–23]. Since the STarT Back Tool is limited to patients with LBP, the need for a single, more generic prognostic stratification tool has been noted. In response to this need, the Keele STarT MSK tool was developed [24]. The STarT MSK tool has shown moderate to good predictive ability in the identification of patients who will develop persistent disabling pain and has also demonstrated good validity for use among primary care patients with the five most common musculoskeletal pain presentations (back, neck, shoulder, knee or multisite pain) [17, 19]. The STarT MSK tool shows acceptable measurement properties and has been translated to several languages: Dutch [25], German [26], Hebrew [27], Norwegian [28] and Persian [29]. The translation and adaptation of a tool such as the STarT MSK can be of value for patients and clinicians globally but also in research, as it allows researchers to investigate how the stratification of patients to various treatment levels can predict outcomes [24]. Moreover, the usefulness of the tool for stratification and treatment matching is important in the effort to improve healthcare. As the STarT MSK tool has not yet been adapted to Swedish, the objective of this study was to translate and cross-culturally adapt the STarT MSK from English into Swedish and to assess the test–retest reliability and construct validity in patients with MSK pain problems seeking primary health care.

## Methods

### Design

This study collected consecutive data from four primary health care physiotherapy clinics situated in urban and suburban Stockholm. In Sweden, patients can seek physiotherapy care with direct access or through a referral from a general practitioner. The study was conducted in two steps: the first step involved the translation and cross-cultural adaption of the STarT MSK from English to Swedish in accordance with international guidelines [30]. The second step involved the evaluation of the instrument's face and construct validity along with the test–retest reliability. Written informed consent to participate in the study was obtained for all participants. The study was approved by the Regional Ethical Review Board in Stockholm and Karolinska Institutet to which ethical approval belongs (ethical approval, Dnr. 2018/2655–32).

### Study population

Data was collected by 1–2 physiotherapists at each clinic. The physiotherapists collecting the data were informed about the study protocol by one of the authors (ERB) before the study started.

Ninety-nine consecutive patients seeking care for MSK disorders (neck, back, knee, hip, multi-pain) were included. If eligible, they were asked by the physiotherapist to participate in the study and gave their written informed consent. Inclusion criteria were men and women 18–70 years of age seeking physiotherapy care with a primary complaint of a MSDs of the neck, shoulder, lower back, hip or knee, or multi-site pain. Exclusion criteria were malignant and inflammatory disorders and the inability to understand spoken and written Swedish.

The study sample size was based on recommended quality criteria for investigating the measurement properties of instruments, which suggests a minimum of 50–100 participants for test–retest reliability and construct validity [31].

### The STarT MSK tool

**Fig. 1:**
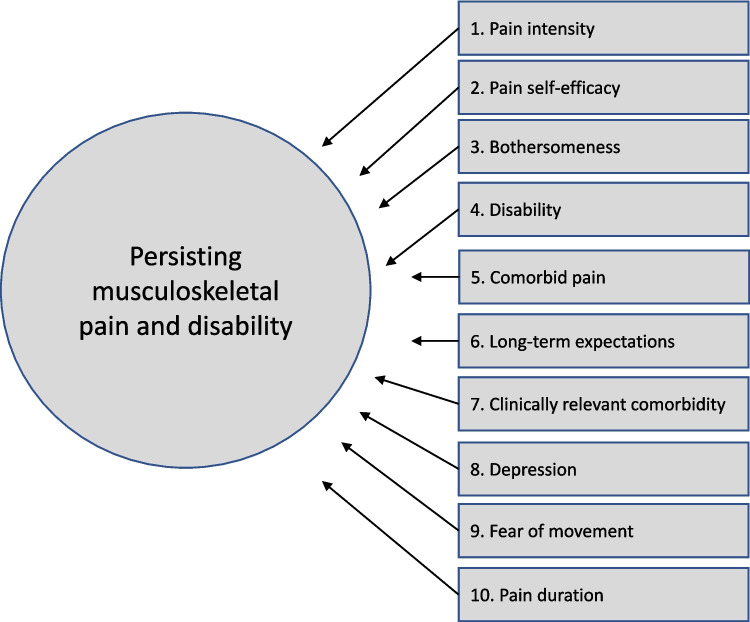
The STarT Musculoskeletal tool including its ten constructs

STarT MSK is a self-report formative tool comprising 10 questions that measure different constructs of functional and psychosocial pain-related factors to predict persistent disabling pain and disability [19] (Fig. 1). The first question is an 10-point numeric rating scale (NRS) to measure pain intensity over the last 14 days and is calculated as zero (0) points if assessed as 0–4, as one (1) point if assessed with 5–6, two (2) points if assessed as 7–8 and, three (3) points if assessed with 9–10. Questions 2–10 are dichotomously answered (yes/no). Thus, a total score of 0–12 points is obtained. The patient is stratified based on the total score to a subgroup with low (0–4), medium (5–8) and high (9–12) risk of persistent disabling pain [32].

### Translation and cross-cultural adaptation

The original STarT MSK was forward and backward translated and cross-culturally adapted to Swedish following international guidelines [30]. Two native Swedish translators, one expert (advanced physiotherapist and researcher) and one naïve, both fluent in English, completed a forward translation from English to Swedish. The two translations were pooled into one translation after consensus discussions in an expert group comprising a researcher in physiotherapy, a physiotherapist with a postgraduate degree in manual therapy and a researcher in public health. Two worked part time or full time with patients suffering from MSDs. The translated version was back-translated from Swedish to English by two other translators who were fluent in Swedish and were native English speakers. Neither had a medical background, nor did they have any prior knowledge of the original version of the STarT MSK tool or the objectives of the study. The expert group consolidated all versions and reached a consensus on discrepancies concerning semantic and conceptual equivalence between the source and target version of the questionnaire and developed the pre-final version of the Swedish STarT MSK.

### Test of the pre-final version

Using the pre-final version, fifteen patients were consecutively included exclusively for the purpose of face validity. In a one-to-one meeting with a physiotherapist at a primary health care clinic, the patients were asked to verbalize their thoughts while completing the pre-final version of the STarT MSK. The physiotherapist encouraged the participants to share their thoughts on their ability to understand the instrument and if they considered that the wording was ambiguous or hard to understand and thus needed to be changed [33].

### Data collection

All participants completed the STarT MSK tool, the reference instruments, and socio-demographic variables (Table 1). For the purpose of this study, three reference instruments were chosen: the modified Örebro Musculoskeletal Pain Questionnaire (ÖMPSQ) [34, 35], the EuroQol 5-dimension (EQ-5D-5L) [36] and the Musculoskeletal Health Questionnaire (MSK-HQ) [37]. The instruments were chosen based on the fact that they measure the same or similar constructs and were used in previous validation studies of the STarT MSK.

**Table 1:** Baseline characteristics of included participants (*n* = 99) and the risk groups low (*n* = 28), medium risk (*n* = 60) and high risk (*n* = 11)

**Variables**	**Total sample** (*n* = 99)	**Low risk** **(*n* = 28)**	**Medium risk** **(*n* = 60)**	**High risk** **(*n* = 11)**
Gender, women, n (%)	66 (66)	17 (61)	41 (68)	8 (73)
Age, years, mean (SD)	49 (14)	49 (12)	49 (14)	45 (14)
Pain site, n (%)				
- Low back	24 (24)	8 (29)	13 (21)	3 (27)
- Neck	14 (14)	3 (11)	10 (17)	1 (9)
- Shoulder	17 (17)	7 (25)	10 (17)	0 (0)
- Hip	10 (10)	0 (0)	7 (12)	3 (27)
- Knee	27 (27)	9 (32)	15 (25)	3 (27)
- Multi-site	7 (7)	1 (4)	5 (8)	1 (9)
Pain duration, months, mean (SD)	20 (48)	17 (48)	21 (53)	21 (21)
NPRS last 7 days median (IQR)	6 (3–7)	4 (3–5)	5 (3–7)	6 (6–8)
Days/week with activity, (0–7), median (range)	3 (2–5)	5 (1–7)	3 (2–5)	3 (0–4)
Total STarT MSK score (0–12), median (IQR)	6 (4–8)	4 (3–4)	7 (6–7)	9 (9–10)
Total ÖMPQ Score median (IQR)	56.5 (43–64)	32 (27–38)	43 (37–50)	57 (52–63)
Total EQ-5D Score (-0.285–1.0) Median (IQR)	0.69 (0.60–0.77)	0.77 (0.72–0.80)	0.68 (0.60–0.73)	0.55 (0.50–0-63)
Total Score MSK-HQ (0–56), median (IQR)	33 (26–38)	36 (34–44)	31 (26–38)	23 (20–27)

*NPRS* Numeric Pain Rating Scale, *EQ-5D* EuroQul 5 dimensions, *ÖMPSQ* Örebro Musculoskeletal Pain Questionnaire, *MSKHQ* Musculoskeletal Health Questionnaire, *SD* Standard Deviation, *IQR* Interquartile range

### Reference instruments

#### The Örebro Musculoskeletal Pain Questionnaire

The modified Örebro Musculoskeletal Pain Questionnaire (ÖMPSQ) includes 10 questions covering the duration of the pain period and questions related to psycho-social risk factors in musculoskeletal pain: self-perceived function, pain experience, fear avoidance beliefs, distress and return to work expectancy. The 10 questions are scored on a scale from 0–10, with a total score of 100 representing the highest risk. A cut-off of ≥ 50 has been suggested to indicate a higher risk group. The Swedish version of the short version of the ÖMPSQ has been validated for non-acute musculoskeletal pain [38].

#### The EuroQol 5-dimension

The EQ-5D-5L measures quality of life and comprises five dimensions: mobility, self-care, usual activities, pain/discomfort, and anxiety/depression and is scored from one (1) (worst imaginable health) to 5 (best imaginable health). The scores are transformed into an index valued ranging from -0.59 to 1, with a score of 1 indicating perfect health [36].

#### The Musculoskeletal Health Questionnaire (MSK-HQ)

The MSK-HQ comprises 14 items aimed to assess the musculoskeletal health status in patients suffering from MSK disorders. It is a formative instrument including aspects shown to be relevant in musculoskeletal health, including questions on pain, fatigue, physical function, sleep, self-efficacy, and psychological well-being [37]. The MSK-HQ includes 14 questions that are scored between 0 and 56, with a higher score representing better health. In addition, the MSK-HQ includes a question on the number of physically active days per week (0–7). The MSK-HQ has shown good reliability and validity in subjects with a range of musculoskeletal disorders [37]. The instrument is translated and validated in a Swedish primary health care context with good test–retest reliability (total score ICC_2.1_ 0.90) and moderate to high construct validity (*r* > 0.30) (unpublished data, the authors).

#### Floor and ceiling effects

Floor (best status) and ceiling (worst status) effects were evaluated and considered to be present if more than 15% of the patients reported the highest or the lowest possible score [39].

#### Test–retest reliability

We investigated the test–retest reliability with an interval of 7–10 days [40]. The first test (T1) was carried out at the physiotherapy clinic. Consecutive participants (*n* = 52) who were also included in the validity part of the study were given a pre-paid envelope at the first test occasion containing the STarT MSK instrument and a question on global change for the second test (T2) (“worse”, “not changed” or “improved”). A reminder was sent by text-message to fill in and mail the follow-up questionnaire seven days after baseline. A second reminder was sent after three more days.

For the total score of the STarT MSK, the Intra Class Correlation (ICC_2.1_) was used with a two-way random effects model to analyse the test re-test reliability. ICC can range from 0 to 1, and values were considered good if ICC was 0.60–0.80 and excellent if > 0.80 [31, 41].

To assess the degree of agreement for each item on repeated measurements, Cohen’s kappa (κ) coefficient was used for items 2–10 [31, 41], and for the first item, a weighted Kappa was used. Kappa can be interpreted as κ < 0.1 = poor; κ: 0.1– 0.2 = slight; κ: 0.21–0.40 = fair; κ: 0.41–0.6 = moderate; κ: 0.61–0.8 = substantial and κ: 0.81–1 = almost perfect [42]. Only participants who were considered stable between the first and second measurement were included in the analysis: those who answered “not changed” on a question on global change (worse, not changed, changed) and those who scored the same on the NPRS plus or minus one point compared with the first measurement test as proposed in other validation studies of STarT MSK [25, 43].

#### Construct validity

Construct validity is by the COSMIN group defined as “the degree to which the scores of an instrument are consistent in relation to predefined hypotheses based on the assumption that the instrument validly measures the construct that is to be measured” [31]. We hypothesised that the total score of the STarT MSK would show a correlation, at least moderate (r ≥ 0.3) with the total score of the included reference instruments. In addition, the 10 separate items from the STarT MSK tool were tested for convergent/divergent validity according to pre-determined questions from the reference instruments to assess the specific constructs with a hypothesis to correlate at least moderately (*r* ≥ 0.3, *r* ≤ 0.3) (Table 3). This method is in concordance with previous validations studies of the STarT MSK [25, 28]. The construct validity was considered acceptable if at least 75% of the predefined hypotheses were fulfilled [31]. Spearman’s Rank correlation coefficients (r) were used in all correlation analyses due to the variety in scale types. The coefficients were described as low (< 0.3), moderate (0.3–0.6) and high (> 0.6) [31].

#### Known-groups validity

The risk groups were described using the cut-off values from the STarT tools for low (0–4), medium (5–8) and high risk (9–12). To investigate the known-groups validity, that is, how the three risk groups are discriminated from each other, a higher sum score (≥ 10%) was expected to be found on the NPRS and the ÖMPSQ and a lower score on the EQ5D and the MSK-HQ (≥ 10%) when comparing the low to the medium, and the medium to the high-risk group [28]. The a-priori hypothesis was that the STarT MSK should be able to differentiate between the three risk groups based on the participants’ answers.

## Results

Table 1 shows the demographics, the results of the STarT MSK and the reference instruments for the total group and the risk groups. A total of 99 consecutive participants were included in the study, with a gender distribution of 68% female. A wide range of pain duration was observed among the participants, with a mean of 20 months and range up to 240 months (20 years). Most participants reported pain in the lower back (*n* = 24) or knee (*n* = 27), and the same pattern was observed in the three risk groups. Seven participants reported multi-site pain. The cohort were stratified into low (*n* = 28), medium (*n* = 60) and high risk (*n* = 11).

### Translation

The STarT MSK was successfully translated from English into Swedish. The testing of the pre-final version revealed minor changes, which did not change the final version. No changes of the Swedish version of the STarT MSK tool were thus made following the pre-final test.

### Floor and ceiling effects

Few (*n* = 2) of the participants scored a minimum on the total score while none scored the maximum score, thus no floor or ceiling effect was shown.

### Test–retest reliability

Forty-nine (*n* = 49) completed the Swedish STarT MSK at the first and the second test. Three (*n* = 3) included in the first test did not answer the second measurement. Thirty-one (*n* = 31) reported “no change” between test one and test two, were considered stable, and were thus included in the analysis. The total score showed a good test–retest reliability (ICC_2.1_ 0.85). The median value for the test–retest of the specific items was Kappa 0.54, and the specific items varied from slight (item 2) to substantial (item 1 and 5) (Table 2).

**Table 2:** Test–retest reliability of the Swedish STarT MSK total score (0–12) and the separate items in stable (unchanged) participants (*n* = 31)

Item	*Kappa*	CI 95%	PA%	CI 95%	SEM
1	0.83	0.68–0.97	94	90–99	0.07
2	0.18	-0.26–0.44	58	36–76	0.18
3	0.37	0.03–0.70	70	54–88	0.16
4	0.53	0.21–0.85	77	62–93	0.16
5	0.87	0.68–1	94	84–1	0.09
6	0.67	0.30–1	90	73–1	0.17
7	0.76	0.48–1	90	73–1	0.13
8	0.49	0.16–0.80	74	58–90	0.15
9	0.54	0.23–0.86	77	62–93	0.15
10	0.67	0.40–0.95	84	70–98	0.13
Total score (*n* = 31)^a^	0.85	0.69–0.93			

*PA* Proportion of Agreement, *CI* Confidence Interval, *SEM* Standard error of measurement

^a^Calculated with ICC_2.1_

### Construct validity

In line with the set hypotheses, the Swedish version of the STarT MSK showed a moderate to high correlation (*r* ≥ 0.30) with all the reference instruments: ÖMPQ (*r* = 0.61), EQ-5D (*r* = -0.59) and the MSK-HQ (*r* = -0.56) (Table 3). The separate STarT MSK items (1–10) showed a correlation with the reference questions, varying between poor to moderate. All but two items (item 2, pain self-efficacy; item 9, fear of movement) were in line with the set hypotheses (*r* ≥ or ≤ 0.3) (Table 3).

**Table 3:** Correlations between the Swedish STarT MSK total score and its separate items and the reference measures

#	Start MSK	Construct	Comparator, item (#) no	Expected R	Estimated R	Correlation	Hypothesis met
	Total score	Persistent disabling pain	ÖMPSQ	*r* ≥ 0.3	0.61	High	Yes
	Total score	Persistent disabling pain	EQ-5D	*r* ≥ 0.3	0.59	Medium	Yes
	Total score	Persistent disabling pain	MSK-HQ	*r* ≥ 0.3	0.56	Medium	Yes
1	Item 1	Pain Intensity	Pain intensity (NPRS)	*r* ≥ 0.3	0.75	High	Yes
2	Item 2	Pain self-efficacy	MSK-HQ #13 Symptom management	*r* ≥ -0.3	-0.21	Low	**No**
3	Item 3	Bothersomeness	MSK-HQ #14 Bothersomeness	r ≥ -0.3	-0.35	Moderate	Yes
4	Item 4	Disability	EQ-5D #1 Mobility	r ≥ 0.3	0.65	High	Yes
5	Item 5	Comorbid pain	ÖMPSQ #8 Return to work	r ≤ -0.3	-0.05	Low	Yes
7	Item 6	Long-term expectations	ÖMPSQ #7 Pain persistency	r ≥ 0.3	0.43	Moderate	Yes
8	Item 7	Clinically relevant comorbidity	EQ-5D #3 Usual activities	r ≤ -0.3	-0.12	Low	Yes
9	Item 8	Depression	EQ-5D #% Anxiety, depression	r ≥ 0.3	0.71	High	Yes
10	Item 9	Fear of movement	ÖMPSQ #9 Fear avoidance	r ≥ 0.3	0.25	Low	**No**
11	Item 10	Pain duration	ÖMPSQ #1 pain duration	r ≥ 0.3	0.77	High	Yes

*NPRS* Numeric Pain Rating Scale, *EQ-5D* EuroQul 5 dimensions, *ÖMPSQ* Örebro Musculoskeletal Pain Questionnaire, *MSK HQ* Musculoskeletal Health Questionnaire

### Known-groups validity

Based on the cut-off values for low, medium, and high risk using the STarT MSK instrument, 28 participants were in the low-risk group (0–4 points), 60 were in the medium risk group (5–8 points) and 11 were in the high-risk group (9–12 points). According to the a-priori hypothesis, the results from the STarT MSK and the reference instruments were able to discriminate between the risk groups by 10% based on how the participants answered (Table 1).

## Discussion

Our aim was to translate, cross-culturally adapt and to test the STarT MSK tool for test–retest reliability and aspects of validity in a Swedish primary health care context. Following the translation into Swedish, the STarT MSK was found to have a substantial test–retest reliability and showed a moderate to high correlation with the selected reference instruments as hypothesized. When considering the total score and the specific items, more than 75% of the pre-defined hypotheses were confirmed in the validation analyses. In the known-groups validity test, the Swedish version of the tool was also able to discriminate between the risk group based on how the participants answered.

The STarT MSK has previously been translated and adapted into Dutch, German, Hebrew, Norwegian and Persian [25–29] with acceptable measurement properties in line with our results. As proposed, we aimed to include at least 50 participants in the test–retest part of the study [31]. However, we included only the participants who were stable between the first and the second measurement, that is, who did not report a global change or showed a change on the NPRS of only one (1) point in concordance with the criteria proposed by Bier et al. [43] and the criteria used in the Dutch validation study [25]. This might be considered too few participants and thus a limitation to the study. Not all previous validation studies investigated the test–retest reliability, and only the German and the Hebrew studies reported the test–retest reliability of the specific items [26, 27]. It might be discussed if it is of value to investigate the separate items for test–retest reliability in addition to the total score. Still, we decided to investigate this to further explain the test–retest reliability.

A challenge when testing the tools validity in comparison to other reference instruments is that the STarT MSK tool is a formative tool, meaning that each item contributes to a specific construct or domain and the specific items collectively give a score, suggesting a prognosis for chronic musculoskeletal disability [17]. It is therefore difficult to validate the score according to a full reference instrument that only measures one construct, for example, an instrument for general health. We used the Swedish version of the ÖMPQ short version, designed to detect those with a risk (> 50 points of 100) for long term pain and disability, which also is considered a formative instrument covering aspects that are shown to be important in musculoskeletal pain [35]. In addition, the Swedish version of the Musculoskeletal Health Questionnaire (MSK-HQ) was used, which also is based on a formative model and, includes questions on various constructs related to musculoskeletal health [37]. The MSK-HQ is however suggested for use in a rehabilitation context for baseline and follow-up, but not for stratification purposes. Importantly, the Swedish version of the MSK-HQ was investigated in a Swedish primary health context with very good measurement properties, but as the results are not yet published (the authors, unpublished data), this might be considered a limitation of the present study.

When analysing how the separate items of the STarT MSK correlated to the reference questions, two of the items did not confirm the pre-determined hypothesis: item 2 in pain self-efficacy and item 9 in fear of movement. One reason the hypothesis was not fully confirmed may be that the items on fear of movement and self-efficacy were phrased in a different way in the STarT MSK compared to the reference instruments, thus capturing the construct in a different way. Our results are partly in line with the those of the Dutch and the Norwegian validation studies which raises the question if these two questions measure the construct intended [25, 28].

The stratification of patients to the separate risk groups was 28%, 66% and 11% to the low-, medium- and high-risk groups, respectively. For the known-groups validity, the a-priori hypothesis of a difference of 10% in how the participants scored in the risk groups was valid for the STarT MSK and for all reference instruments. This means that the Swedish version of the STarT MSK tool seems to be able to discriminate between different levels of musculoskeletal factors. To analyse known-groups validity, a minimum of 50 participants per group is recommended. We aimed to include a minimum of 100 participants as this is recommended in validation studies [31]. Since we did not include 50 participants per risk group, the results of the known-groups validity should therefore be interpreted with caution. However, what strengthened our results is that all our instruments still were able to differentiate between the three risk groups. Evens so, the value of a stratification in relation to targeted treatment and thus the outcome after treatment needs to be further investigated for the Swedish version.

Strengths of the current study include our adherence to international recommendations for translation, the test–retest reliability, and for the validation part of the study [30–32]. We investigated the construct validity, following the COSMIN taxonomy based on hypothesis testing, where > 75% of our hypothesis were confirmed [31, 32, 44]. Furthermore, we included the recommended number of participants for the test-reliability and validation parts, even though the included number still can be considered low compared to other validation studies, for example the Norwegian study [28]. Our participants who were consecutively included at four primary health care clinics can, however, be considered representative of those seeking primary care for musculoskeletal pain.

We did not include all aspects of measurement properties as recommended by the COSMIN group [32]. The STarT MSK is built on a formative model, meaning that the ten items included in the tool measure separate constructs as described in Fig. 1, hence the items are not expected to be correlated. Therefore, an analysis of internal consistency or to explore the structural validity of the tool was not considered relevant [43, 44]. It can, nonetheless, be debated how a total score of a scale such as the STarT MSK tool is used [45]. The purpose of STarT MSK is to screen for risk of persistent disability and not for follow-up purposes. Therefore, to investigate the responsiveness is not essential [44]. The total score of the STarT MSK tool is used to stratify the patient into one of three sub-groups, where each point represents a prognostic factor considered important for persistent disability with the higher score meaning a higher risk [18]. One very important aspect of validity, though not included in the present study, is the predictive validity to identify if the stratification to risk groups is successful in terms of targeted care. The original STarT MSK Tool has reported good predictive ability, identifying patients at low, medium or high risk of persistent musculoskeletal pain over 6-months [19] in line with the Dutch, the German and the Hebrew validation studies [25, 26, 46]. The predictive validity of the Swedish version of the STarT MSK tool is therefore planned to be investigated in a future project. Given the findings of the present study we, however, believe that the Swedish version of the STarT MSK tool is useful in a primary health context to support clinicians to better target patients suffering from MSK complaints so that they receive an adequate level of care.

## Conclusion

The STarT MSK has been successfully translated and adapted into Swedish and shows acceptable measurement properties regarding test–retest reliability and aspects of validity and seems to be able to discriminate between the proposed risk groups. A future study needs to determine the tools predictive validity and to investigate if stratification to risk groups leads to a faster recovery and to lower health care costs.

## Data Availability

The datasets generated and/or analysed during the current study are not publicly available due to ethical regulations at Karolinska Institute but are available from the corresponding author upon reasonable request.
